# A Novel Relative High-Density Lipoprotein Index to Predict the Structural Changes in High-Density Lipoprotein and Its Ability to Inhibit Endothelial–Mesenchymal Transition

**DOI:** 10.3390/ijms22105210

**Published:** 2021-05-14

**Authors:** Feng-Yen Lin, Yi-Wen Lin, Chun-Ming Shih, Shing-Jong Lin, Yu-Tang Tung, Chi-Yuan Li, Yung-Hsiang Chen, Cheng-Yen Lin, Yi-Ting Tsai, Chun-Yao Huang

**Affiliations:** 1 Taipei Heart Institute, Taipei Medical University, Taipei 110, Taiwan; g870905@tmu.edu.tw (F.-Y.L.); cmshih53@tmu.edu.tw (C.-M.S.); sjlin@tmu.edu.tw (S.-J.L.); 2 Department of Internal Medicine, School of Medicine, College of Medicine, Taipei Medical University, Taipei 110, Taiwan; 3 Division of Cardiology, Department of Internal Medicine and Cardiovascular Research Center, Taipei Medical University Hospital, Taipei 110, Taiwan; 4 Institute of Oral Biology, National Yang Ming Chiao Tung University, Hsinchu 300, Taiwan; ywlin@ym.edu.tw; 5 Graduate Institute of Biotechnology, National Chung Hsing University, Taichung 238, Taiwan; peggytung@nchu.edu.tw; 6 Department of Anesthesiology and Graduate Institute of Clinical Medical Science, China Medical University and Hospital, Taichung 406, Taiwan; cyli168@gmail.com; 7 Graduate Institute of Integrated Medicine, China Medical University, Taichung 406, Taiwan; yhchen@mail.cmu.edu.tw; 8 Department of Psychology, College of Medical and Health Science, Asia University, Taichung 413, Taiwan; 9 Healthcare Information and Management Department, Ming Chuan University, Taoyuan 333, Taiwan; a684094@ms28.hinet.net; 10 Division of Cardiovascular Surgery, Tri-Service General Hospital, National Defense Medical Center, Taipei 115, Taiwan

**Keywords:** high-density lipoprotein, endothelial progenitor cells, endothelial-to-mesenchymal transition, relative high-density lipoprotein index

## Abstract

Therapeutic elevation of high-density lipoprotein (HDL) is thought to minimize atherogenesis in subjects with dyslipidemia. However, this is not the case in clinical practice. The function of HDL is not determined by its concentration in the plasma but by its specific structural components. We previously identified an index for the prediction of HDL functionality, relative HDL (rHDL) index, and preliminarily explored that dysfunctional HDL (rHDL index value > 2) failed to rescue the damage to endothelial progenitor cells (EPCs). To confirm the effectiveness of the rHDL index for predicting HDL functions, here we evaluated the effects of HDL from patients with different rHDL index values on the endothelial–mesenchymal transition (EndoMT) of EPCs. We also analyzed the lipid species in HDL with different rHDL index values and investigated the structural differences that affect HDL functions. The results indicate that HDL from healthy adults and subjects with an rHDL index value < 2 protected transforming growth factor (TGF)-β1-stimulated EndoMT by modulating Smad2/3 and Snail activation. HDL from subjects with an rHDL index value > 2 failed to restore the functionality of TGF-β1-treated EPCs. Lipidomic analysis demonstrated that HDL with different rHDL index values may differ in the composition of triglycerides, phosphatidylcholine, and phosphatidylinositol. In conclusion, we confirmed the applicability of the rHDL index value to predict HDL function and found structural differences that may affect the function of HDL, which warrants further in-depth studies.

## 1. Introduction

Similar to hypertension, diabetes mellitus, smoking, and aging, hypercholesterolemia is a major risk factor for cardiovascular disease [1]. Alleviation of low-density lipoprotein (LDL) level has always been the focus of cardiovascular disease treatment and prevention regimens. High-density lipoprotein (HDL) performs several anti-atherosclerotic functions [2]. Its surface contains protein particles with different properties. These protein particles enable HDL to regulate the ability of tissues to absorb cholesterol and maintain lipid balance in the body by promoting the release and recovery of excess cholesterol [3]. During reverse cholesterol transport, HDL removes excess cholesterol from atherosclerotic plaques. In addition, HDL may increase endothelial nitric oxide (NO) production, reduce oxidative stress, lower inflammatory responses, decrease endothelial adhesion protein expression, inhibit monocyte migration, and infiltrate the sub-endothelial space [4,5]. However, recent studies have shown that the structures related to anti-atherosclerotic formation in HDL particles are transformed into pro-inflammatory and harmful substances in response to viral and bacterial infections [5] and type 2 diabetes mellitus [6]. Therefore, the function of HDL is not determined by the cholesterol content of the particle but by its specific structural components. Current studies focus not on the mere concentration of HDL in the blood but on its actual function.

The Norfolk case–control study and Incremental Decrease in End Points through Aggressive Lipid Lowering (IDEAL) study have demonstrated that enhanced plasma levels of HDL-C (≥70 mg/dL), including large particles of HDL (>9.53 nm), increase the risk of coronary artery disease [7]. As very large HDL particles are rich in cholesterol, functionally defective HDL assumes the role of a cholesterol provider rather than that of a scavenger and promotes the process of atherosclerosis [8]. In another study, people with HDL containing more cholesterol molecules were shown to progress faster toward carotid atherosclerosis than those with HDL containing lower cholesterol molecules [9,10]; extremely high HDL-C levels are associated with hyperlipidemia and atherosclerosis-induced mortality [11,12]. These studies have shed light on the negative impact of dysfunctional HDL (also called defective HDL) on human physiology. Research on defective HDL was originally derived from the observation of patients with lupus erythematosus (SLE). According to statistics, the incidence of cardiovascular disease in these patients is 5–50 times higher than that in healthy population [13], while 47% of female patients with SLE or 27% of women with rheumatoid arthritis (RA) have defective HDL [14]. In addition, patients with acute coronary syndrome have more defective HDL content in the blood than those with stable angina [15]; HDL isolated from patients with coronary arterial disease shows pro-inflammatory effects rather than anti-inflammatory properties when exposed to endothelial cells [16] because of its inhibitory effects on endothelial nitric oxide synthase activity and production of NO, leading to the loss of anti-inflammatory capability [17].

Given the discovery of defective HDL, the use of plasma HDL level to evaluate the function of HDL and estimate the incidence of atherosclerosis has been questioned. We believe that the effectiveness of HDL against atherosclerosis must be investigated to predict the incidence of cardiovascular disease in the clinic, and develop related drugs. Scientists currently believe that “the number of circulating endothelial progenitor cells (EPCs)” and “their clone formation ability” can be used as indicators of vascular repair capacity and vascular health. In this direction, we used flow cytometry to detect the number of circulating EPCs and performed in vitro cultures to analyze their cloning ability in the circulation to evaluate their functions. In addition, we performed the 2,7-dichlorodihydrofluorescein (DCF) assay to analyze the antioxidant capacity of HDL from patients. We preliminarily calculated an index (defined as the relative HDL index, rHDL index) based on the value derived from an enzyme immunofluorescence assay. The number of EPCs in circulation and the clone formation ability of EPCs are significantly lower for those with an rHDL index value ≥ 2 than for subjects with an rHDL index value < 2.We speculate that the defined x value ≥ 2 may determine the quality of HDL and clinically evaluate its functional status [18], although several studies are still underway to confirm the credibility and availability of this indicator.

EPCs can migrate to the lesion for adhesion and differentiation [19], repair the damaged area, and inhibit the occurrence of atherosclerosis. Factors such as oxidative stress [20,21], blood sugar [22], hypoxia [23], and inflammation [24] can facilitate differentiation of EPCs into expected endothelial cells following migration to the injury site of the endothelium; however, EPCs can also differentiate into vascular smooth muscle-like cells (VSMCs) [25] through endothelial-to-mesenchymal transition (EndoMT) [26].

Previous studies conducted in the model of animal bone marrow transplantation found that cells differentiated from bone marrow will thicken the arterial intima and increase the formation of atherosclerotic plaques [27,28]. Therefore, scientists believe that the EPCs that can repair vascular endothelium migrate to the injury site but differentiate into smooth muscle cells-like phenotype cells [29]. Whether EPCs can perform their functions at the injured site remains a concern for many scientists [27,28]. EndoMT can be induced by transforming growth factor-beta 1 (TGF-β1) [30]. TGF-β1 activates intracellular transcription factors (snail, slug, smad and zeb1 and zeb2) in the process of initiating EndoMT in endothelial cells (ECs) [31,32], reduces the expression of specific proteins (VE-cadherin, vWF) on the surface of ECs, and increases the performance of interstitial proteins (α-SMA, myocardin, vimentin, FSP-1) expression. These changes causevascular ECs to have the cellular characteristics of myofibroblast-like cells and migratory phenotype [25], and snail, especially, plays an extremely important role. In addition, endothelin-1 (ET-1) increases the migration and inflammatory response of cells on the vessel wall, and it also stimulates EPCs [25,33]. IL-1b and oxLDL can enhance EndoMT of ECs induced by TGF-β1 [34] or by radiation [35]. EndoMT plays important roles in the course of heart failure, kidney diseases, atherosclerosis etc. [36,37]. EndoMT triggered by TGF-β1 is affected by several inflammatory factors [37,38]. However, there are fewer studies on the effects of lipid profile on EndoMT in EPCs.

In comparison with the effect of healthy HDL, that of defective HDL on EndoMT is yet unknown [39]. Therefore, in this study, we analyzed the effects of HDL from patients with different rHDL index values on the EndoMT of isolated EPCs and investigated the difference in lipoprotein content in HDL. We look forward to further confirm the reliability of the rHDL index value (using 2 as a cut-off point) for evaluating the anti-atherosclerotic effects of HDL.

## 2. Results

### 2.1. TGF-β1 Induces Morphological Changes and EndoMT in EPCs That May Be Inhibited by HDL from Patients with rHDL Index Value Lower Than 2

**Figure 1:**
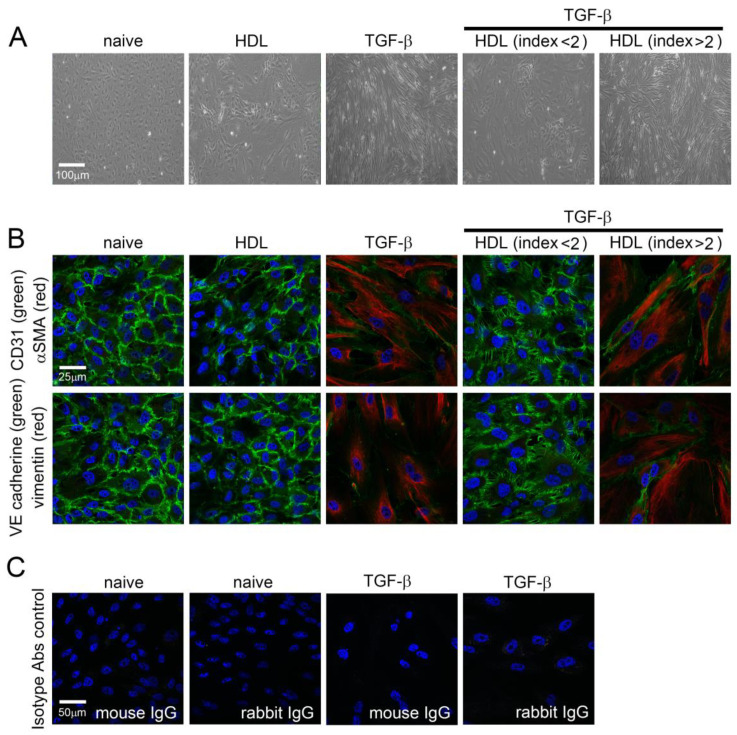
TGF-β1-induced morphological changes and EndoMT of EPCs are inhibited by HDL from patients with an HDL index < 2. (**A**) Human EPCs grown on slides were exposed to 10 ng/mL recombinant human TGF-β1 for 5 days with or without HDL (400 μg/mL HDL from healthy volunteer, patient with an HDL index < 2, or patient with an HDL index > 2) treatment. The morphology of EPCs was observed using light microscopy under 100× magnification. (**B**) Experimental EPCs were subjected to immunofluorescence, and imaged under a confocal microscope. The expression of α-SMA (red), vimentin (red), CD31 (green), and VE cadherin (red) was determined using specific antibodies. The sections were counterstained with Hoechst 33258 to identify the nucleus. (**C**) The naïve and TGF-β1-treated EPCs were stained with isotype mouse control IgG or rabbit control IgG as negative control, respectively. Images at 400× magnification are shown.

As EndoMT of EPCs plays a critical rolein the development of vascular disease and is induced by TGF-β1, we verified the direct effect of HDL on EndoMT of TGF-β1-induced EPCsin vitro. To confirm that human EPCs are responsible for TGF-β1 and HDL treatment, cell morphological characteristics were observed by light microscopy (Figure 1A). Naïve EPCs were spindle shaped and evenly distributed at confluence. After 5 days of treatment with HDL from healthy volunteers, EPCs were morphologically similar to naïve cells. The cells became slender, tightly packed, and directional after stimulation with 10 ng/mL TGF-β1 for 5 days, and this phenomenon was inhibited by treatment with 400 μg/mL HDL from patients with an rHDL index < 2. In contrast, the HDL from patients with an rHDL index value > 2 did not rescue the morphological changesin cells under TGF-β1 stimulation. As shown in Figure 1A, immunofluorescence stainingwas performed to identify EndoMT-related protein expression. The results demonstrated high CD31 and VE-cadherin expression (green signal) and low α-SMA and vimentin expressionin naïve and healthy HDL-treatedEPCs. Stimulation with 10 ng/mL TGF-β1for 5 days induced α-SMA and vimentin expression (red) and decreased CD31 and VE-cadherin expression (green) in EPCs. CD31- and VE-cadherin-positive cells were present inEPCs stimulated with TGF-β1 in the presence of healthy HDL as compared with that in the EPCs stimulated with TGF-β1 alone (Figure 1B). In contrast, CD31- and VE-cadherin-positive cells were not observed in the group treated with HDL from patients with an rHDL index value >2. These results indicate that TGF-β1-induced EndoMTof EPC may be rescued by HDLextracted from patients with an rHDL index value <2.

### 2.2. TGF-β1-Induced Expression of EndoMT-Related Genes Was Regulated by HDL from Healthy Subjects and Patients with Lower rHDL Index

**Figure 2:**
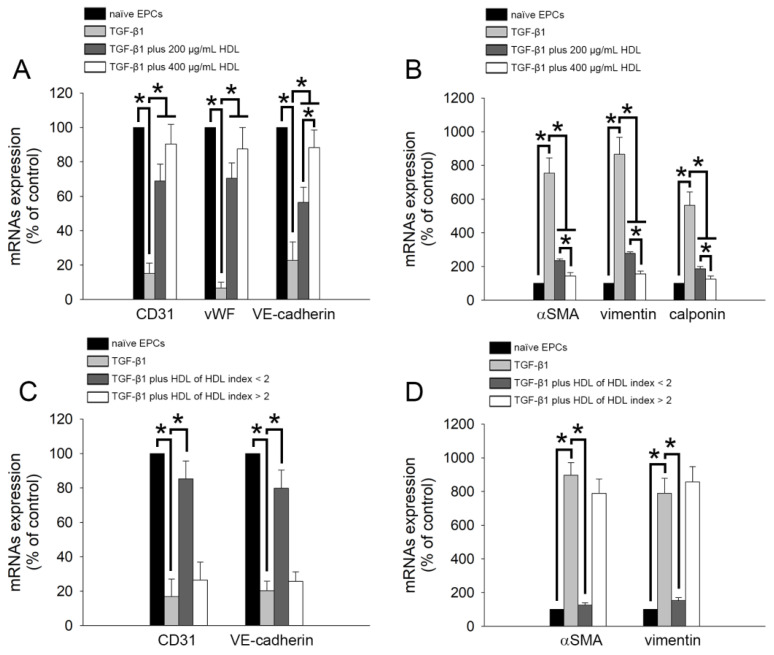
HDL can inhibit the TGF-β1-mediated EndoMT, and this effect is determined by the quality of HDL. (**A**,**B**) Human EPCs were exposed to 10 ng/mL recombinant human TGF-β1 for 5 days without or with HDL (200 or 400 μg/mL HDL from healthy volunteers) treatment (black: naïve EPCs; light grey: TGF-β1; dark grey: TGF-β1 plus 200 μg/mL HDL; white: TGF-β1 plus 400 μg/mL HDL). The mRNA expression of the genes encoding CD31, vWF, VE-cadherin, α-SMA, vimentin, and calponin was evaluated using reverse transcription and qPCR analysis. (**C**,**D**) Human EPCs were exposed to TGF-β1 for 5 days with or without HDL (400 μg/mL HDL from patient with an HDL index < 2 or patient with an HDL index > 2) treatment (black: naïve EPCs; light gray: TGF-β1; dark gray: TGF-β1 plus HDL of HDL index < 2; white: TGF-β1 plus HDL of HDL index > 2). The mRNA expression of the genes encoding CD31, vWF, VE-cadherin, α-SMA, vimentin, and calponin was identified by reverse transcription and qPCR analysis. Five patient samples were used, and five independent experiments were performed (*n* = 5). The related mRNA expression (normalized to GAPDH mRNA expression) is presented as a bar graph. All data are expressed as the Mean ± Standard Deviation of five independent experiments and as the percentage of the control. Statistical evaluations were performed using the one-way ANOVA followed by Dunnett’s test. * *p* <0.05 was considered significant.

To further analyze the effect of HDL on the EndoMT of TGF-β1-stimulated EPCs, we performed reverse transcription and qPCR to analyze the expression of related genes (CD31, vWF, VE-cadherin, α-SMA, vimentin, and calponin). As shown in Figure 2A, treatment with 10 ng/mL TGF-β1for 5 days decreased the expression of the genes encoding CD31, vWF, and VE-cadherin (CD31 mRNA: 15.2% ± 5.9% of control; vWF mRNA: 6.7% ± 3.4% of control; VE-cadherin mRNA: 22.7% ± 10.7% of control) as compared with the control treatment (naïve group). Co-treatment with HDL from healthy volunteers reversed the downregulation of the expression of EndoMT-related genes, although no dosage-dependent effect was observed at concentrations between 200 and 400 μg/mL on CD31 and vWF gene expression (CD31 mRNA: 68.9% ± 9.8% of control in 200 μg/mL HDL treatment group and 90.4% ± 11.4% of control in 400 μg/mL HDL treatment group; vWF mRNA: 70.5% ± 8.9% of control in 200 μg/mL HDL treatment group and 87.5% ± 12.5% of control in 400 μg/mL HDL treatment group; VE-cadherin mRNA: 56.4% ± 8.8% of control in 200 μg/mL HDL treatment group and 88.3% ± 10.2% of control in 400 μg/mL HDL treatment group). We also analyzed the expression of smooth muscle cell-related genes (α-SMA, vimentin, and calponin). Figure 2B demonstrates that TGF-β1 stimulation significantly increased the mRNA expression of the genes encoding α-SMA (768.6% ± 81.6% of control), vimentin (857.9% ± 99.8% of control), and calponin (598.7% ± 69.8% of control). Consistent with the results shown in Figure 1A, 200 and 400 μg/mL HDL treatment effectively reduced EndoMT (decreased α-SMA, vimentin, and calponin expression at the mRNA level in a dose-dependent manner) in EPCs treated with TGF-β1 (Figure 2B). In addition, we analyzed the impact of HDL from patients with different rHDL indices on the expression of EndoMT-related genes. As shown in Figure 2C,D, 400 μg/mL HDL from patients with lower rHDL index value (<2),but not from those with higher rHDL index value, increased the mRNA expression of the genes encoding CD31 and VE-cadherin and decreased the expression of the genes encoding α-SMA and vimentin. Thus, the HDL-mediated inhibition of the EndoMT induced by TGF-β1 depends on its quality (rHDL index value can be used as an indicator of HDL quality) rather than its concentration.

### 2.3. TGF-β1-Induced EndoMT Was Regulated by HDL from Healthy Subjects and Patients with Lower rHDL Index

**Figure 3:**
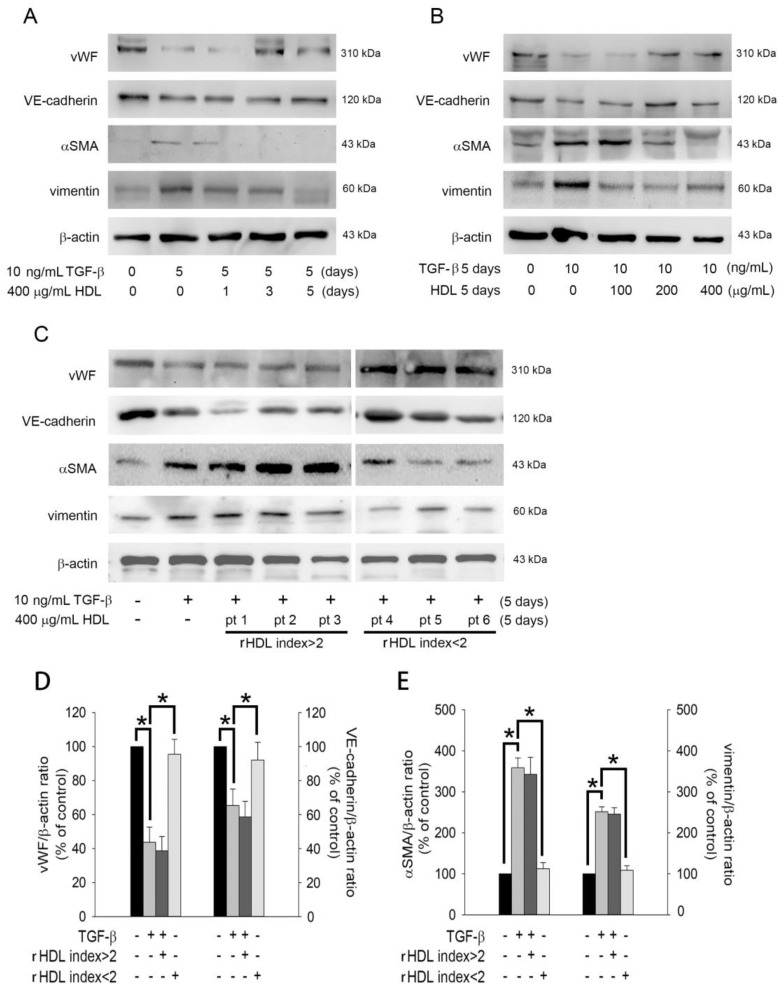
The poor quality of HDL from patients with an HDL index < 2 may affect its inhibitory effect on EndoMT. (**A**) Human EPCs were treated with 10 ng/mL recombinant human TGF-β1 for 5 days in the presence or absence of healthy 400 μg/mL HDL for 1–5 days. (**B**) Human EPCs were treated with 10 ng/mL recombinant human TGF-β1for 5 days with or without 100–400 μg/mL HDL for 5 days. (**C**) Human EPCs were treated with 10 ng/mL recombinant human TGF-β1 for 5 days with or without 400 μg/mL HDL (from patients with an HDL index >2 or <2) for 5 days. Total cell lysates were purified, and the levels of vWF, VE-cadherin, SMA, and vimentin were analyzed using western blotting; β-actin was used as a loading control. (**D**,**E**) The density of each band was quantified using densitometry, and related protein expression is presented as vWF/β-actin ratio, VE-cadherin/β-actin ratio, α-SMA/β-actin ratio, or vimentin/β-actin ratio. Bar graph (Mean ± Standard Deviation) is presented as percentage of control. The one-way ANOVA followed by Dunnett’s test was used for statistical analysis, and * *p*< 0.05 was considered significant.

To confirm the impact of HDL on the EndoMT of TGF-β1-stimulated EPCs, we performed western blotting to analyze the expression of related proteins (vWF, VE-cadherin, α-SMA, and vimentin). As shown in Figure 3A, treatment with 10 ng/mL TGF-β1 for 5 days decreased the expression of vWF and VE-cadherin proteins as compared to the control treatment (naïve group). Co-treatment with 400 μg/mL HDL from healthy volunteers could ameliorate the expression of vWF (3 and 5 days) and VE-cadherin (5 days). In contrast, TGF-β1significantly increased α-SMA and vimentin expression, which was significantly prevented by healthy HDL in 1–5 days. Figure 3B demonstrates that HDL from healthy donors may reverse EndoMT in a dose-dependent manner. In contrast, only HDL from patients with an rHDL index value < 2 (patients 4–6), but not HDL from patients with an rHDL index value > 2 [patients 1–3]) could inhibit EndoMT (by upregulating vWF and VE-cadherin expression and downregulating α-SMA and vimentin expression). In summary, HDL from healthy donors inhibited the EndoMT of EPCs induced by TGF-β1in a dose-dependent manner. However, this effect would diminish if the quality of HDL is poor (HDL from patients with an rHDL index value < 2).

### 2.4. HDL Regulates TGF-β1-Induced EndoMT via Smad- and Snail-Dependent Pathways

**Figure 4:**
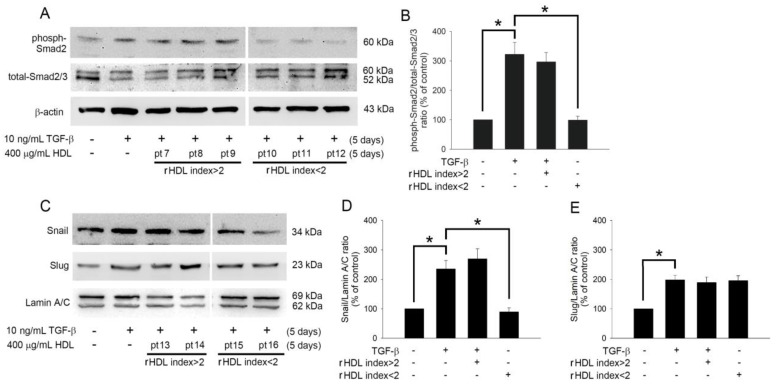
HDL regulates the TGF-β1-induced EndoMT via Smad- and Snail-dependent pathways. (**A**) Human EPCs were treated with 10 ng/mL recombinant human TGF-β1 for 5 days without or with 400 μg/mL HDL (from patients with an HDL index >2 or <2) for 5 days. Total cell lysates were purified, and the levels of phosphorylated Smad2 and total Smad2/3 were analyzed using western blotting; β-actin was used as a loading control. (**C**) Human EPCs were treated with TGF-β1in the presence of absence of 400 μg/mL HDL (from patients with an HDL index >2 or <2) for 5 days. Total nuclear lysates were purified, and the levels of Snail and Slug were analyzed using western blotting; lamin A/C was used as a loading control. (**B**,**D**,**E**) The density of each band was quantified using densitometry, and related protein expression was presented as phoph-Smad2/totalSmad2/3 ratio, Snail/Lamin A/C ratio, or Slug/Lamin A/C ratio. Bar graphs (Mean ± Standard Deviation) are presented as percentages of the control. Statistical evaluations were performed using one-way ANOVA followed by Dunnett’s test, and * *p*< 0.05 was considered significant.

TGF-β1 induces phosphorylation and activation of Smad2/3, which is associated with EndoMT. Therefore, the effects of HDL on Smad2/3 activation in TGF-β1-treated EPCs were analyzed by western blotting. Figure 4A demonstrates that HDL from patients (7 to 9) with an rHDL index value > 2 could not inhibit Smad2 phosphorylation. However, HDL from patients (10 to 12) with an rHDL index value < 2 inhibited Smad2 activation in TGF-β1-stimulated EPCs. Snail and Slug are transcriptional factors that positively regulate the expression of EndoMT markers [40] and mediate the loss of cell-cell adhesion in endothelial cells [41]. Therefore, we analyzed the impact of HDL on Snail and Slug activation. As shown in Figure 4C, TGF-β1 induced the activation of Snail and Slug, and the HDL from patients (15 and 16) with an rHDL index value < 2 inhibited the nuclear translocation of Snail; however, this effect was not observed in the presence of HDL from patients (13 and 14) with an rHDL index value > 2. Regardless of its type (higher or lower rHDL index), HDL had no effect on the activation of Slug in TGF-β1-induced EPCs. Based on these results, we conclude that HDL from patients with low rHDL index values can inhibit EndoMT by controlling the phosphorylation of Smad2/3 and activation of Snail transcription factors in EPCs; however, the role of other signaling pathways cannot be neglected. On the contrary, HDL has no effect on the activation of the Slug signaling pathway known to regulate EndoMT.

### 2.5. The Clinical Characteristics Did Not Differ between Patients with rHDL Index> and <2.

To understand the difference in the lipid profiles of HDL from the two groups (rHDL index value >2 and <2), we performed lipidomic analysis for 20 HDL samples from patients (10 patients for rHDL index value > 2 group and10 patients for rHDL index value < 2). We collected blood samples, isolated HDL, and performed the DCF assay to identify rHDL index, which was used to group patients. Table 1 presents the clinical characteristics of the patients. There were five female and five male patients in each group. The age, body weight, and body height of the patients presented a normal distribution. Patients with an rHDL index value > 2 were not significantly different from those with an rHDL index value < 2 in terms of age, body weight, and body height. The incidence of hypertension and diabetes mellitus did not significantly differ between the two groups. None of the participants had smoked or abused alcohol in the preceding 6 months. Furthermore, none had previously experienced peripheral vascular disease, chronic obstructive pulmonary disease, stroke, myocardial infarction, autoimmune disorders, rheumatoid arthritis, asthma, chronic kidney deficiency, and cancers.

### 2.6. HDL from Patients with rHDL Index Value > 2 Had Higher Relative Abundance of Glycerolipid in Select Triglyceride (TG), Phosphatidylcholine (PC), and Phosphatidylinositol (PI) Species

**Figure 5:**
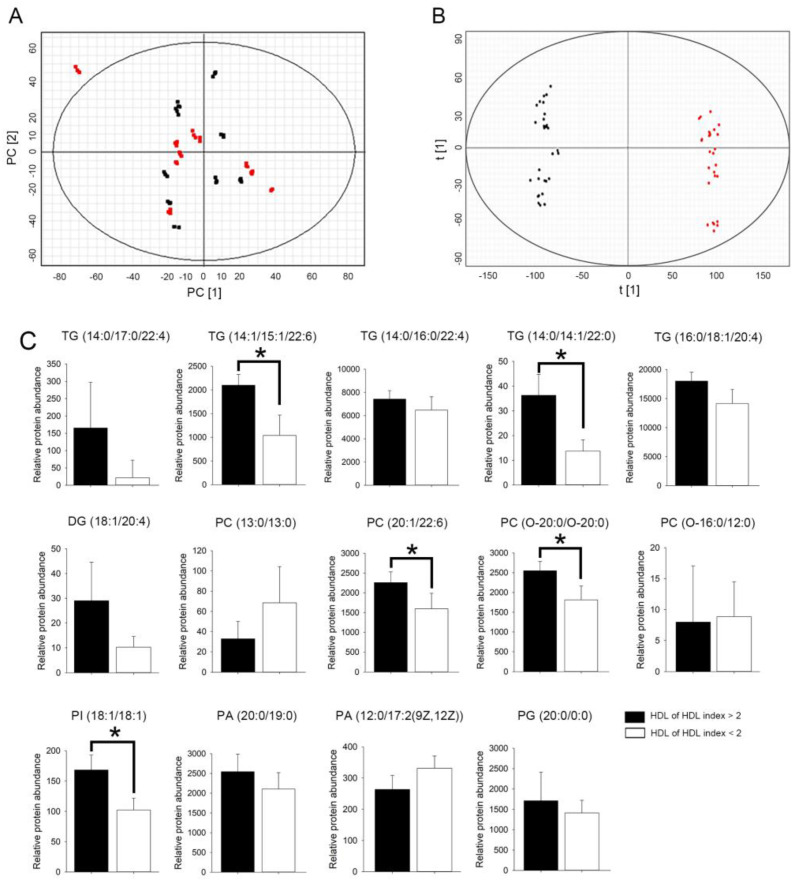
Score plot of the metabolic profile of HDL from patients with an HDL index less or more than 2. (**A**) Principal component analysis (PCA) score plot of the metabolic profile of HDL from patients with an HDL index > 2 (black dot) versus HDL index > 2 (red dot). (**B**) OPLS-DA score plot of lipid profile of HDL from patients with an HDL index > 2 (black dot) versus HDL index > 2 (red dot) after unit variance scaling. (**C**) Bar graphs showed the relative abundance of TG, DG, PC, PI, PA, and PG species in patients with an rHDL index value > (black bar) or <2 (white bar). Statistical evaluations were performed using the Student’s t-test followed by Mann-Whitney U test. Results are presented as Mean ± Standard Deviation; a * *p* < 0.05 was considered statistically significant.

We used UPLC-QTOF/MS to analyze the lipid composition of HDL. HDL lipid profiles, including 68 lipid species in the positive-ion mode and 324 lipid species in the negative-ion mode, were selected by nontargetedlipidomics from a total of 20 HDL samples. Principal component analysis (PCA) score plots obtained for patients from the two groups are shown in Figure 5. PCA revealed a cloudy separation between patients with rHDL index values >2 and < 2 (Figure 5A). However, as shown in Figure 5B, the OPLS-DA score plot revealed a clear separation between the two groups with good fitting and predictive performance (R^2^Y = 0.815; Q^2^ = 0.792).

We subsequently explored and identified potential lipid biomarkers of HDL. The lipid species features with variable importance in projection (VIP) value > 1.0, fold change (FC) > 2.0, and ANOVA *p* < 0.05 were considered potentially different lipids species. As shown in Table 2, 14 specific lipid species could distinguish the rHDL index value > 2 group from the rHDL index value < 2 group. TG, diglyceride (DG), PC, PI, phosphatidic acid (PA), and phosphatidylglycerol (PG) were significantly upregulated in the HDL from the rHDL index value > 2 group as compared to that in the HDL from the rHDL index value < 2 group; this result should be further externally validated by lipidomic analysis. HDL from patients with an rHDL index value > 2 showed higher abundance of glycerolipid species than the HDL from patients with an rHDL index value < 2. The selected TG (TG [14:0/14:1/22:0]) and TG (TG [16:0/16:0/16:0]), PC (PC [20:1/22:6]), and PC (PC [O-20:0/O-20:0]), and PI (PI [18:1/18:1]) species showed higher relative abundance in the rHDL index value > 2 group than in the rHDL index value < 2. Therefore, the lipid species in the structure of HDL from patients with different rHDL index values will be different. We speculate that this may be a critical factor for the difference in HDL function, although the roles of TG, PC, and PI in the HDL structure are currently unclear.

## 3. Discussion

The density of HDL is high because of its high proportion of proteins. HDL has a monolayer membrane structure formed by phospholipids, free cholesterol, and apolipoprotein to coat esterified cholesterol particles [42]. Apolipoprotein is a smooth spherical lipoprotein particle that can bind and transport cholesterol by interacting with the cell surface. Approximately 30% cholesterol in the body is transported by HDL [43]. The biosynthesis of HDL mainly occurs in the liver and small intestine. HDL comprises small disc-shaped particles containing low lipids and larger spherical particles rich in cholesterol. Thus, several complex particles such as lipids, proteins, polypeptides, and sterols make up HDL.HDL can be further classified according to its physical and chemical properties, such as density, shape (disc or spherical), or type of apolipoprotein.

HDL assists the metabolism of cholesterol in the blood by transporting it to the liver, a process termed as “cholesterol efflux”. In addition, the apolipoprotein of HDL can help infiltrated macrophages in the arterial wall to expel out cholesterol, thereby inhibiting foam cell accumulation and atherosclerotic formation; this effect is mediated by ATP-binding cassette subfamily A member 1(ABCA1) on macrophages that can promote unidirectionally flow of cholesterol to Apo-A1, ABCG1, and scavenger receptor class B type 1 (SR-B1) of unsaturated lipids and assist its removal from HDL [44]. Previous proteomic analysis of HDL revealed its unexpectedly complex composition. There are 200 different apolipoproteins distributed in various HDL subgroups that are essential for the reverse transport of cholesterol [45]. In a complex metabolic process, HDL can also be used as a medium for material transportation. It can carry and transport proteins, vitamins, non-coding RNA, and microRNAs to remote organs [46]. Impairment in these complex functions leads to several diseases. The cholesterol efflux capacity of macrophages can be considered as an important indicator of HDL function [47,48,49]. Many clinical studies have shown that the occurrence of cardiovascular events is related to the cholesterol efflux ability dominated by HDL but not to HDL concentration in the blood [47,50]. We believe that the evaluation of HDL function from the reverse transport of cholesterol and the cholesterol efflux capacity is an appropriate strategy for predicting the risk of cardiovascular events. However, the applicability of these two indicators to predict the probability of future cardiovascular risk has not been confirmed because the outflow of cholesterol in the body is easily affected by the environment [51]. In other words, the total outflow of cholesterol measured in the test tube experiment may not accurately simulate the transfer of cholesterol between cells and tissues in the body. Given that the function of HDL may be related to the heterogeneity of its particles (such as the content of specific apolipoprotein and glycerolipid), accurate analysis and understanding of the structural variation of HDL may allow effective evaluation of its functions.

EPCs play a very important role in the repair of the vascular endothelium and the proliferation of blood vessels during hypoxia. As “number of circulating EPCs” and “colony formation capacity of EPCs” can be used as indicators of vascular repair capacity and vascular health, we used the content and function of EPCs in the blood as indicators and performed the DCF assay to analyze the functional status of HDL [18]. We defined a new index called the rHDL index; HDL from patients with an rHDL index value < 2 could effectively reverse the damage of oxLDL to EPCs, while HDL from patients with an rHDL index value > 2 had no such effect. This poorly functioning HDL can be called dysfunctional HDL. The high rHDL index value of dysfunctional HDL may not only imply its poor antioxidant performance but also indicate its inability to protect EPCs. A side from oxLDL-induced atherosclerosis, oxHDL is also found to be a risk factor [52,53]. The production of dysfunctional HDL is caused by a mutation or deletion of an apolipoprotein-encoding gene of HDL [54,55]. However, similar to LDL, HDL is also easily oxidized, especially when modified by acrolein [56]. As oxLDL isolated from the patient’s plasma contains the same proportion of oxHDL particles, researchers believe that the interaction between LDL and HDL occurs during the oxidative modification of LDL, and that both LDL and HDL will contribute to atherosclerosis [53]. HDL is hypothesized to be involved in the transfer and metabolism of PC derivatives. There are many different modifications to lipoproteins, of which oxidation of polyunsaturated fatty acids is known to cause oxidation of apolipoprotein and lipids [57,58]. Oxidized phospholipids in lipoproteins can regulate the biological functions of lipoproteins, such as induction of proliferation and cytokine production of macrophages and vascular smooth muscle cells [59]. The phospholipid changes in lipoproteins can be identified by UPLC-QTOF/MS [60]. Lyso-PC was shown to spontaneously transfer from LDL to HDL through the action of lecithin-cholesterol acyltransferase (LCAT) [61]. In addition, short-chain oxPC can also be transferred between lipoproteins and easily hydrolyzed by lipoprotein-associated phospholipase A2 (Lp-PLA2), suggesting that dysfunctional HDL can participate in the oxidation process of LDL [61]. In the present study, we found that HDL from patients with an rHDL index> 2 did not have the ability to inhibit the EndoMT of EPCs, and exhibited changes in the TG, PC, and PI composition, which may indirectly or directly reduce the EPC population in the blood, decrease EPC function, and prevent inhibition of TGF-β1-induced EndoMT. This is the first study to demonstrate that the structural differences in HDL may be the cause of its functional differences. Future studies will explore the effect of structural changes in HDL on its functions and the mechanism underlying the effect of variant structure on cell function.

## 4. Materials and Methods

### 4.1. Ethics and Patient/Volunteer Demographics

The Taipei Medical University-Joint Institutional Review Board approved this study (TMU-JIRB No.: 201302008 and No.: N201711082) in 26 June 2019, and informed consent was obtained from fivehealthy volunteers (20–24 years old) or people who visited our cardiology center for dyslipidemia consultation (total 20 patients were collected). Patients were excluded from the study if they had autoimmune problems, rheumatoid joint inflammation, asthma, chronic kidney shortage, persistent obstructive pulmonary condition, cancer, stroke, prior heart attack, and/or peripheral artery disease. In addition, those who had received steroidal or nonsteroidal anti-inflammatory medicines, Chinese herbal medicines or extraction, andlipid-reducing therapies such as statins, fibrates, niacin, or ezetimibe within 1 year prior to this study were also excluded.we performed lipidomic analysis for 20 HDL samples from patients (10 patients for rHDL index value > 2 group and10 patients for rHDL index value < 2). We collected blood samples, isolated HDL, and performed the DCF assay to identify rHDL index, which was used to group patients. Table 1 presents the clinical characteristics of the patients. There were five female and five male patients in each group.

### 4.2. Preparation of HDL

The HDL fraction was isolated from the human serum and characterized as previously described [62,63]. Briefly, the plasma was collected from the blood withdrawn into 0.38% sodium citrate tubes. The major lipoprotein classes of HDL (d = 1.063–1.210 g/mL) were prepared by sequential ultracentrifugation. The density was adjusted with solid sodium bromide (NaBr) and extensively dialyzed at 4 °C for 24 h in phosphate-buffered saline (PBS, 5 mM phosphate buffer and 125 mM sodium chloride [NaCl], pH 7.4). In our experiments, the extent of oxidation was monitored by measuring thiobarbituric acid reactive substances (TBARS) and by horizontal electrophoresis. To identify minor contamination (e.g., HDL oxidation during HDL storage) that may also result in impaired EPC function, horizontal electrophoresis and TBARS assays were used to analyze the components and oxidation state of HDL before each study. To avoid possible effects of heavy metal contamination in HDL samples, isolated HDL was dialyzed at 4 °C for 24 h in PBS containing 0.3 μM EDTA. To avoid contamination with endotoxins, aseptically isolatedlipoproteins were tested using ToxinSensor™ Chromogenic LAL Endotoxin Assay Kit (GenScript, Piscataway, NJ, USA). The concentration of lipoprotein was determined using Bradford Protein Assay Kit (ThermoFisher Scientific, Waltham, MA, USA). In this study, only HDL prepared within 7 days was used.

### 4.3. EPC Isolation and Cultivation

Mononuclear cells were separated from volunteers, and EPCs were extracted by density-gradient centrifugation using Histopaq-1077 (density 1.077 g/mL; Sigma-Aldrich, Burlington, MA, USA). The Taipei Medical University Institutional Review Board accepted this research study (No.: 201302008 in 29 July 2018), and all participants provided written informed consent to participate in this research. The protocols for EPC seclusion and growth have been explained previously [64].

### 4.4. Immunofluorescence Staining

EPCs seeded on cover slips (4 × 10^4^) were treated under the experimental conditions. After washing once, the cells were fixed with 4% paraformaldehyde and permeabilized for 10 min with a 0.1% Triton X-100 solution. Nonspecific antigens were blocked with 2% bovine serum albumin for 30 min, and the slides were then double-stained with mouse anti-α-smooth muscle actin (α-SMA; Sigma-Aldrich, Burlington, MA, USA) and mouse anti-hCD31 (ThermoFisher Scientific, Waltham, MA, USA) antibodies or rabbit anti-vimentin (GeneTex, Alton Pkwy Irvine, CA, USA) and rabbit anti-von Willebrand factor (vWF; Chemicon, CA, USA). Alexa 594-conjugated antibody was used to detect anti-α-SMA and anti-vimentin antibodies, and Alexa 488-conjugated antibody was used to detect anti-CD31 and anti-vWF antibodies. Nuclei were identified using Hoechst 33258 (Sigma-Aldrich, St. Louis, MO, USA). Images were obtained using a Zeiss LSM 700 confocal microscope (Carl Zeiss MicroImaging Inc., Thornwood, NY, USA).

### 4.5. Real-Time Quantitative Polymerase Chain Reactions

Total RNA was isolated using the TRIzol reagent kit (Invitrogen, Waltham, MA, USA), according to the manufacturer’s instructions. Reverse transcription and quantitative real-time polymerase chain reaction (PCR) were performed. The levels of CD31, vWF, VE-cadherin, calponin, α-SMA, and vimentin mRNA expression were determined in arbitrary units by comparison with an external DNA standard amplified by the vWF, CD31, calponin, and α-SMA primers, respectively. The PCR primers used for cDNA amplification are presented in Table 3.

### 4.6. Western Blot Analysis

Total cell lysates and membrane proteins were processedaspreviously reported [65]. Protein concentrations in the supernatants were measured using a Bio-Rad protein determination kit (Bio-Rad, San Jose, CA, USA). The supernatants were subjected to 8% or 10% sodium dodecyl sulfate polyacrylamide gel electrophoresis (SDS-PAGE) and transferred to polyvinylidene difluoride membranes for 1 h at room temperature. The membranes were treated for 1 h at room temperature with PBS containing 0.05% Tween−20 and 2% skimmed milk and separately incubated for 1 h at room temperature with primary antibodies. Mouse anti-VE-cadherin and rabbit anti-hvWF antibodies were purchased from Millipore Co. (Billerica, MA, USA). Mouse anti-hα-SMA and mouse anti-vimentin antibodies were supplied by Sigma-Aldrich (San Diego, CA, USA). Mouse anti-β-actin antibody was procured from Santa Cruz Biotechnology (Santa Cruz, CA, USA), and rabbit anti-phosphorylated Smad2, anti-total Smad2/3, anti-Snail, and anti-Slug antibodies were obtained from Cell Signaling Co. (Danvers, MA, USA). Membranes were then incubated with a horseradish peroxidase-conjugated IgG. Immunodetection was performed using a chemiluminescence reagent following exposure to a ChemiDoc-ItTM Imaging System (UVP, Upland, CA, USA). A densitometer was used for quantitation, and the results are shown as percentage of the control.

### 4.7. DCF Assay for Identification of rHDL Index Value

The rHDL index value of HDL from healthy and balanced individuals was determined according to our previous study. Lipid oxidation products may hydrolyze 2,7-dichlorofluorescin diacetate (DCFH-DA) to DCF, which produces extreme fluorescence. Therefore, the antioxidative capability of HDL was evaluated using the DCF assay [66]. In brief, DCFH-DA in methanol (2.0 mg/mL) was added to 1-palmitoyl-2- arachidonoyl-sn-glycero-3-phosphoryl-choline (PAPC) in chloroform (2.0 mg/mL) as well as hydroperoxyoctadeca-9Z, 11 E-dienoic acid (HOPE) in ethanol (0.1 mg/mL) and vortexed. The mixture was treated with HDL and incubated at space temperature at night for 2 h to identify the bioactivity of HDL by avoiding oxidation of PAPC plus HPODE. The fluorescence intensity was determined using a spectrofluorometer at excitation and emission wavelengths of 485 and 530 nm, respectively. The higher the fluorescence intensity, the lower is the antioxidative capacity of HDL.

### 4.8. Lipidomic Analysis

In brief, 1.5 mL methanol was added to 40 µL HDL sample, and the mixture was incubated with 3 mL of chloroform for 1 h at room temperature with periodic vortexing. Later, 1.25 mL of distilled water was added, and the mixture was allowed to stand for 10 min to facilitate phase separate on. The sample was centrifuged at 1000× *g* for 10 min at 4 °C, and aliquots of 2 mL of the natural stage were collected. Finally, the aliquots were vacuum-dried and stored at −80 °C until further analysis.

HDL was reconstituted in 250 μL isopropanol-acetonitrile-water (2:1:1), and SYNAPT G2 QT (Waters MS Technologies, Manchester, UK) was used for Ultra-high performance liquid chromatography-quadrupole time-of-flight mass spectrometry (UPLC-QTOF/MS) analysis. The parameters of the mass spectrometer for positive-ionization mode detection were as follows: desolvation gas flow, 900 L/h at 550 °C; cone gas flow, 15 L/h; source temperature, 120 °C; capillary voltage, 2.8 kV; cone voltage, 40 V; and TOF-MS scan range, 100–2000 *m*/*z*. The data acquisition rate was set to 1.2 s with a 0.02 s interscan delay using Waters MSE acquisition mode, and the full exact masses were simultaneously collected by rapidly alternating between two functions. Function 1 acquired data with a low collision energy of 4 and 2 eV for trap and transfer collision cells, whereas function 2 acquired data using a transfer collision energy ramp from 15 to 35 eV. All analyses were performed using LockSpray to ensure accuracy and reproducibility. Leucine-enkephalin was used as the lock mass at a concentration of 1 ng/μL and a flow rate of 5 μL/min. Data were collected in continuum mode, and the LockSpray frequency was set at 20 s. All data acquisition was controlled using Waters MassLynx v4.1 software (Waters MS Technologies, Milford, MA, USA).

### 4.9. Lipid Identification

The raw data were imported into Progenesis QI software (Waters MS Technologies, Milford, MA, USA) for alignment. Furthermore, the peak picking and identification of polar lipids were carried out using high-resolution positive-ion MS, and the absolute intensities of all identified compounds were recalculated to determine the relative abundances and normalize the values of the lipid molecules. The data were then exported into EZ info 2.0 software (Waters MS Technologies, Milford, MA, USA) for multivariate statistical analysis, and principal component analysis (PCA) and partial-least-squared discriminate analysis (OPLS-DA) were used to create the final statistical models to obtain group clusters. Lipid molecules with the strongest effect on group clustering were identified as those with variable importance in projection (VIP) greater than 1. In addition, potential ions with *p* less than 0.05 and fold change (FC) > 2 were selected for further metabolite relationship pathway characterization using the MetaboAnalyst web server. Human Metabolome Database (HMDB) IDs were matched with the Kyoto Encyclopedia of Genes and Genomes (KEGG) IDs for KEGG mapping. IDs without a match were excluded from the analysis, and the human pathway library in the KEGG database was selected for analysis.

### 4.10. Statistics Analysis

Values are expressed as the Mean ± Standard deviation. Statistical evaluations were performed using the Student’s *t*-test followed by Mann-Whitney U test or one-way analysis of variance (ANOVA) followed by Dunnett’s test. Results with a *p* value of <0.05 were considered statistically significant.

## 5. Conclusions

Analysis of total cholesterol, TG, LDL, and HDL levels in the plasma to predict incidence of cardiovascular events and effects of drug treatment is now obsolete. In addition to reducing the content of total cholesterol, TG, and LDL in the body, the current anticipation of drugs for the treatment of dyslipidemia is to correct the functional deficiency of HDL, including reduction in the content of dysfunctional HDL and increment in the content/activity of functional HDL. Along with the continuous progress and development of drugs, it is imperative to specifically analyze the texture and structural changes in HDL, understand the effects of these changes on its function, and effectively evaluate and quantify its activity. The breakthrough in the modern analysis technology provides information on the structural changes in various lipids, chylomicrons, glycerolipids, and phospholipids. Therefore, analysis of the structural changes and functions of lipoproteins will become a part of precise medical strategies for cardiovascular diseases. In this study, we confirmed that the rHDL index value score set to 2 can be used as a platform standard for HDL functional analysis, and also verified the impact of HDL from patients with different HDL index backgrounds on TGF-β1-induced EndoMT of EPCs and the underlying signaling pathways. At present, we do not know how the changes in TG (14:1/15:1/22:6), TG (14:0/14:1/22:0), PC (20:1/22:6), PC (O-20:0/O-20:0), and PI (18:1/18:1) affect the function of HDL. We are actively conducting further analyses and hope to establish the importance of accurately evaluating this HDL index to facilitate its application for detecting the efficacy of drugs related to dyslipidemia.

## Figures and Tables

**Table 1:** The clinical characteristics of patients with rHDLindex > or <2.

	Patients with rHDL Index > 2 (*n* = 10)	Patients with rHDL Index < 2 (*n* = 10)
Gender (Male/Female)	5/5	5/5
Age (years)	61.5 ± 5.7	65.9 ± 4.7
Body weight (kg)	63.5 ± 15.7	65.7 ± 19.4
Body height (cm)	167.2 ± 13.7	164.1 ± 19.2
Smoke (*n*, %)	0, 0%	0, 0%
Alcohol drinking (*n*, %)	0, 0%	0, 0%
Hypertension (*n*, %)	8, 80%	9, 90%
Hypercholesterolemia (*n*, %)	10,100%	10,100%
Diabetes mellitus (*n*, %)	2, 20%	3, 30%
Peripheral vascular disease (*n*, %)	0, 0%	0, 0%
Chronic obstructive pulmonary disease (*n*, %)	0, 0%	0, 0%
Old stroke (*n*, %)	0, 0%	0, 0%
Prior myocardial infarction (*n*, %)	0, 0%	0, 0%
Autoimmune disorders	0, 0%	0, 0%
Rheumatoid arthritis	0, 0%	0, 0%
Asthma	0, 0%	0, 0%
Chronic kidney deficiency	0, 0%	0, 0%
Cancer	0, 0%	0, 0%

Values are mean ± SD.

**Table 2:** Statistical analysis of differential lipids to distinguish HDL extracting from rHDL index >2 and rHDL index >2 group.

NO	Formula	Description (Lipid)	Neutral Mass	*m*/*z*	Retention Time (min)	*p* Value	Fold Change	VIP Value
1	C56H100O6	TG(14:0/17:0/22:4)[iso6]	840.721	1779.520	16.068	0.02	4.50	2.12
2	C54H88O6	TG(14:1/15:1/22:6)[iso6]	832.658	853.642	11.413	<0.01	2.19	1.70
3	C55H98O6	TG(14:0/16:0/22:4)[iso6]	854.736	1710.471	16.414	<0.01	2.67	2.08
4	C53H100O6	TG(14:0/14:1/22:0)[iso6]	832.752	1683.528	16.697	<0.01	2.18	1.98
5	C53H98O6	TG(14:0/14:1/22:1)[iso6]	830.734	1679.501	16.414	0.01	2.77	1.82
6	C50H83N3O15P2	DG(18:1/20:4)	1027.530	1060.566	4.086	<0.01	2.37	1.75
7	C34H68NO8P	PC(13:0/13:0)	649.468	970.594	4.107	0.02	2.33	1.73
8	C50H86NO8P	PC(20:1/22:6)	859.609	896.564	10.776	<0.01	4.08	1.7
9	C48H100NO6P	PC(O-20:0/O-20:0)	831.708	1653.485	16.393	0.05	3.15	1.82
10	C36H74NO7P	PC(O-16:0/12:0)	633.502	708.489	2.723	0.02	2.11	1.61
11	C45H83O13P	PI(18:1/18:1)	862.557	1743.145	9.821	<0.01	2.70	2.08
12	C42H83O8P	PA(20:0/19:0)	746.583	1535.212	16.047	0.01	2.13	1.82
13	C32H59O8P	PA(12:0/17:2(9Z,12Z))	602.395	616.413	4.598	0.02	2.14	1.51
14	C26H53O9P	PG(20:0/0:0)	540.343	1098.708	4.148	0.05	2.26	1.8

TG, triglyceride; DG, diglyceride; PC, phosphatidylcholine; PI, phosphatidylinositol; PA, phosphatidic acid; PG, phosphatidylglycerol.

**Table 3:** The primer sequence for real-time RCR.

Gene	Forward Primer	Reverse Primer
CD31	5′-GTG AAG TCC GGA AAG CTG TCC-3′	5′-GGG CAG GTT CAT AAA TAA GTG CAC-3′
vWF	5′-GGC TGC AGT ATG TCA AGG TGG-3′	5′-AGA GCC ATT GGT GCA GTG CAG-3′
VE-cadherin	5′-AGA CAA TGG GAT GCC AAG TCB-3′	5′-AAG ATG AGC AGG GTG ATC ACT G-3′
calponin	5′-ACC TCT ACG ACC CCA AGC TG-3′	5′-GAC ATT GAG CGT GTC GCA GTG-3′
αSMA	5′-CTA TCA GGG GGC ACC ACT ATG-3′	5′-CCG ATC CAG ACA GAG TAT TTG CG-3′
vimentin	5′-AGG CAA AGC AGG AGT CCA CTG A-3′	5′-ATC TGG CGT TCC AGG GAC TCAT -3′
GAPDH	5′-TGC CCC CTC TGC TGA TGC C-3′	5′-CCT CCG ACG CCT GCT TCA CCA C-3′

vWF, von Willebrand factor; αSMA, alpha smooth muscle cell actin; GAPDH, glyceraldehyde 3-phosphate dehydrogenase.

## Data Availability

Not applicable.
